# Detection and characterization of human astrovirus and sapovirus in outpatients with acute gastroenteritis in Guangzhou, China

**DOI:** 10.1186/s12876-021-02044-5

**Published:** 2021-12-03

**Authors:** Xin Luo, Jian-kai Deng, Xiao-ping Mu, Nan Yu, Xiaoyan Che

**Affiliations:** 1 Department of Laboratory Medicine, Guangdong Women and Children Hospital, Guangzhou, 511400 China; 2 Department of Laboratory Medicine, The First Affiliated Hospital, Sun Yat-sen University, Guangzhou, 510080 China; 3 Laboratory of Emerging Infectious Diseases and Division of Laboratory Medicine, Zhujiang Hospital, Southern Medical University, No. 253 Gong-ye Avenue, Guangzhou, 510282 People’s Republic of China

**Keywords:** Acute gastroenteritis, Astrovirus, Sapovirus, Clinical features, Molecular epidemiology

## Abstract

**Background:**

Human astrovirus (HAstV) and sapovirus (SaV) are common pathogens that can cause acute gastroenteritis (AGE). However, very few studies have reported the molecular epidemiology and clinical information on HAstV and SaV in China. This study aims to determine the molecular epidemiology and clinical features of HAstV and SaV in patients with AGE in Guangzhou, China.

**Methods:**

For this study, 656 patients with AGE were enrolled. Their stool samples were screened for 15 enteropathogens using Luminex xTAG^®^ Gastrointestinal Pathogen Panel. HAstV and SaV were detected through an in-house multiplex reverse transcriptase polymerase chain reaction followed by phylogenetic analysis. We described and compared clinical features of AGE in patients with HAstV and SaV.

**Results:**

Of the 656 stool samples, 63.72% (418/656) were found to be positive, with 550 enteropathogens (296 bacteria and 254 viruses). HAstV and SaV were detected in 20 (3.0%) and 12 (1.8%) samples, respectively. Four genotypes (genotypes 1, 2, 3, and 8) of HAstV and three genotypes (GI.1, GI.2 and GIV) of SaV were identified. Coinfection was observed in ten HAstV-positive and two SaV-positive samples. HAstV was more likely to occur in winter, while SaV in early spring. The median age of the patients with single HAstV infection was higher than that of the patients with other viruses (rotavirus, norovirus, and enteric adenovirus; *P* = 0.0476) and unknown etiology (*P* = 0.006). Coinfection with HAstV or SaV were not associated with disease severity (*P* > 0.05).

**Conclusion:**

HAstV and SaV are the common causes of AGE in Guangzhou, China.

## Background

Acute gastroenteritis (AGE) is a common illness of humans globally. It adversely affects the public health, especially the very young, the elderly, the malnourished, and those with an impaired immune system [1–3]. Human AGE is caused by a spectrum of viruses and bacteria. Viruses, including norovirus, rotavirus, enteric adenovirus, astrovirus (HAstV), and sapovirus (SaV), are the major causative agents for AGE [4, 5]. Although detailed epidemiological data for both domestic and overseas infectious gastroenteritis caused by rotavirus, norovirus, and enteric adenovirus is available [6, 7], little is known about the clinical symptoms, characteristics, and coinfection of HAstV- and SaV-related AGE because of the various limitations of the detection methods and low detection rates. Further epidemiological and molecular information about HAstV and SaV causing AGE may prove useful in the development of HAstV/SaV vaccines and other preventative therapies for AGE in the post rotavirus-vaccination introduction era.

The present study aims to determine the incidence of HAstV and SaV in outpatients with AGE in Guangzhou, China, and characterize HAstV and SaV according to their genotype, age-related distribution, seasonal pattern and clinical symptoms.

## Methods

### Sample collection

From September 2013 to January 2016, 656 stool samples were collected from 656 outpatients who were diagnosed with AGE. The definition of AGE was the sudden onset of > 3 episodes of diarrhea or vomiting in the preceding 24 h and symptom duration < 7 days. All of the enrolled stool samples were routinely collected and stored at − 70 °C prior to investigation. The stool samples were preprocessed using NucliSENS easyMAG Lysis Buffer (BIOMERIEUX, France), followed by Viral RNA and DNA extraction using QIAamp MinElute Virus Spin kit (QIAGEN, Germany), as described previously [8].

### Multiplex RT-PCR and nucleotide sequencing

In-house multiplex RT-PCR was developed to detect HAstV, SaV, norovirus, and enteric adenovirus. The primers used for the amplification and sequencing for HAstV (target *ORF1a* gene), SaV (target *ORF1* gene), norovirus GI/GII (target *ORF2* gene), and enteric adenovirus (target *Hexon* gene) were obtained from the published literature [9–12]. The primers used were formulated by Sangon Biotech (Shanghai, China). The product sizes were 482 bp (enteric adenovirus), 288 bp (HAstV), 434 bp (SaV), 330 bp (norovirus GI), and 387 bp (norovirus GII). The PCR was performed on a Veriti PCR (Life Technologies, USA) using the PrimeScript One Step RT-PCR Kit (TaKaRa, Japan). Briefly, 25 μL of the reaction mix comprised Enzyme Mix (1.0 μL), buffer (12.5 μL), RNase-free water (1.0 μL), primers (20 μmol/L each), and RNA/DNA extraction (5.0 μL). Reverse transcription was carried out at 50 °C for 30 min, followed by predenaturation at 94 °C for 3 min, 94 °C 30 s, 54 °C 30 s, and 72 °C 45 s for 40 cycles with a final extension at 72 °C for 12 min. The amplified viral nucleic acid product was detected by 2% agarose gel electrophoresis. Multiplex RT-PCR products that were positive for HAstV and SaV were sequenced by a commercial service (BGI Tech Corporation, Shenzhen, China).

### xTAG gastrointestinal pathogen panel assay

Following the manufacturer’s protocol, the xTAG Gastrointestinal Pathogen Panel (xTAG GPP) assay was used to detect the genes of 15 enteropathogens—*Salmonella* spp.*, Shigella* spp.*, Campylobacter* spp., *Yersinia. enterocolitica*, *Vibrio cholerae, Escherichia. coli* O157, enterotoxigenic *E. coli* (ETEC) LT/ST, Shiga-like toxin-producing *E. coli* (STEC) stx1/stx2, *Clostridium difficile* toxins A/B, rotavirus A, enteric adenovirus 40/41, norovirus GI/GII, *Cryptosporidium* spp., *Entamoeba histolytica*, and *Giardia lamblia.*

### Phylogenetic analysis

The obtained sequences of the HAstV- and SaV-positive samples were aligned by Clustal X (version 5.0). Phylogenetic tree was constructed by the neighbor-joining method using MEGA 6.0 software. The genetic distance was calculated using the Kimura 2-Parameters model with 1000 bootstrap resamples of the nucleotide alignment for genotypic strain classification. The following reference strains and accession numbers were used: L23513, L13745, AF141381, KF039913.1, JQ403108, GQ901902, AF290508, AF260508, FJ973620, GQ502193, FJ402983, GQ502192, NC_019028, and NC_013443 for HAstV; and U65427, X86560, U95643, AB518056, U73124, U95644, AB614356, HM800913, HUN3739, KJ826502, AY603425, AY237420, U95645, AJ249939, AF182760, AY425671, AF435814, DQ125333, and AY64685 for SaV. The nucleotide sequence data reported herein have been published in the GenBank nucleotide sequence database under the accession numbers of HAstV and SaV isolates: KP090035-KP090047, KR364727-KR364729, KX522566-KX522573, KT751010-KT751011, and KX530323-KX530328.

### Statistical analysis

All statistical analyses were performed using SPSS version 21.0 (SPSS Inc., Chicago, IL, USA). Differences among the proportions were compared by the Chi-square Test, Fisher’s Exact Test, or Mann–Whitney U Test. Differences with *P* < 0.05 were considered statistically significant.

## Results

### Distribution of enteropathogens in patients with AGE

A total of 656 stool samples were enrolled in the present study. The age distribution of the patients varied from 9 days to 84 years, with the median age of 25 months. Out of the 656 patients, 387 (59.0%) were male and 521 (79.4%) were under 5 years old. We detected 418 (63.72%) positive samples, resulting in 550 enteropathogens (296 bacteria and 254 viruses). HAstV and SaV were detected in 20 (3.0%) and 12 (1.8%) samples, respectively, while rotavirus was detected in 112 (17.1%), norovirus in 103 (15.7%), and enteric adenovirus in 7 (1.1%) samples. Norovirus GI and GII and adenovirus were detected by both the xTAG GPP and the in-house multiplex RT-PCR (Kappa value was 0.884, 0.918, and 0.791, respectively, showing high consistency between xTAG GPP and the in-house multiplex RT-PCR) [13].

Among people with AGE who tested positive for HAstV, 50% (10/20) had one or more codetected enteropathogens: there are one other pathogen in 40% (8/20), and there are two other pathogens in 10% (2/20). 16.7% (2/12) of SaV positive samples had one or more codetected enteropathogens: there are one other pathogen in 0.2% (1/12), and there are three other pathogens in 0.2% (1/12). Rotavirus, Norovirus, *Clostridium difficile, Salmonella, Campylobacter* were the common codetected enteropathogens. (Table 1).

**Table 1:** Coinfection with HAstV and SaV among people with acute gastroenteritis in Guangzhou, China from 2013 to 2016

Infection or coinfection	No. (%) of AGE cases (n = 656)
HAstV alone	10 (1.5)
SaV alone	10 (1.5)
2 pathogens codetected	
HAstV, rotavirus	3 (0.5)
HAstV, norovirus	3 (0.5)
HAstV, *Clostridium difficile*	1 (0.2)
HAstV, *Salmonella*	1 (0.2)
SaV, norovirus	1 (0.2)
3 pathogens codetected	
HAstV, *Clostridium difficile*, Norovirus	1 (0.2)
HAstV, rotavirus, *Campylobacter*,	1 (0.2)
4 pathogens codetected	
SaV, *Campylobacter*, rotavirus, *Salmonella*	1 (0.2)

### Seasonal and age distribution of HAstV and SaV infection in people with AGE

**Fig. 1:**
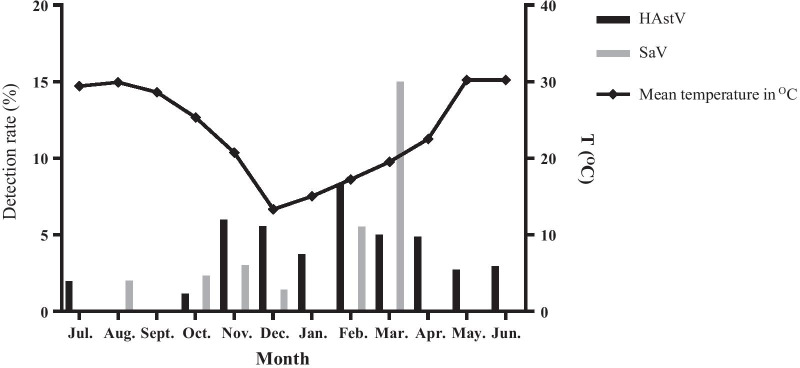
Monthly distribution of HAstV and SaV in people with acute gastroenteritis

Seasonal variation in HAstV and SaV infection is shown in Fig. 1. The monthly mean temperature for the period 2013–2016 in Guangzhou is also shown in Fig. 1. HAstV was detected most commonly in February (8.33%, 3/36). The peak of SaV was March (15.0%, 3/20).

**Fig. 2:**
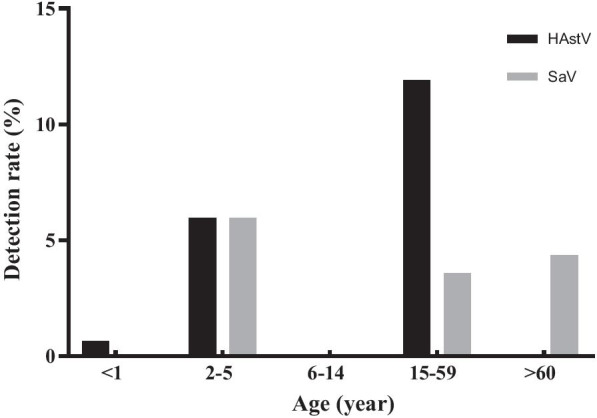
Age distribution of HAstV and SaV in people with acute gastroenteritis

Age distribution in HAstV and SaV infections is shown in Fig. 2. HAstV showed the highest detection rate in 15–59-year age group (11.9%), follow by 1–5-year age group (5.97%). SaV infection was most common in 1–5-year age group (5.97%). HAstV and SaV were not detected in the 5–14-year-old age group. The median age of people with AGE due to single HAstV infection was higher than that of people with other viruses (rotavirus, norovirus, and enteric adenovirus) (*P* = 0.0476) and unknown etiology (*P* = 0.006) (Table 2).

**Table 2:** Comparison of clinical characteristics in the HAstV and SaV infection among people with acute gastroenteritis in Guangzhou, China from 2013 to 2016

Variables	Single HAstV infection (*n* = 10)	Coinfection with HAstV (*n* = 10)	Single SaV infection (*n* = 10)	Coinfection with SaV (*n* = 2)	Other virus infection (*n* = 202)	Unknown etiology cases (*n* = 238)
Demographics						
Male, *n* (%)	6 (60)	6 (60)	3 (30)	2 (100)	124 (61.39)	145 (60.92)
Age range (year)	0.92 to 49	0.03 to 42	1 to 72	1.17 to 2	0.08 to 81	0.08 to 84
Median (IQR)	11.00 (1.00, 27.50)*	15.50 (1.23, 32.75)	2.50 (1.33, 25.00)	1.59 (1.17, 2.00)	1.00 (0.92, 3.00)	0.67 (0.42, 1.00)
Clinical presentations						
Fever > 37.5 °C, *n* (%)	5 (50)**	1 (10)	1 (10)	0 (0)	66 (32.67)	40 (16.81)
Vomit, *n* (%)	2 (20)	5 (50)	2 (20)	0 (0)	97 (48.02)	30 (12.61)
Diarrhea, *n* (%)	10 (100)	10 (100)	10 (100)	2 (100)	202 (100)	238 (100)
Frequency (times/day), median (IQR)	6.50 (4.75, 8.50)	4.50 (3.00, 6.25)	3.50 (3.00, 7.00)	3.00 (3.00, 3.00)	5.00 (4.00, 8.00)	5.00 (4.00, 8.00)
Laboratory findings in stool						
WBC count (/HP), *n* (%)	2 (20)	4 (40)	1 (10)	0 (0)	58 (28.71)	78 (32.77)
OB positive, *n* (%)	4 (40)	2 (20)	2 (20)	0 (0)	49 (24.26)	73 (30.67)

Unknown etiology cases: no enteric pathogens were detected

Other virus infection: includes rotavirus, norovirus, and enteric adenovirus infection, but excludes coinfection with HAstV and SaV

*OB* stool occult blood test

*P < .05 compared to other virus infection and unknown etiology cases

**P < .05 comspared to unkown etiology cases

### Clinical features of HAstV and SaV infections in people with AGE

Single SaV infections and infections by other viruses or unknown etiology did not have much different clinical features such as fever, vomit, diarrheal frequency, and stool routine examination (WBC count and OB text). However, AGE with single HAstV infection is more likely to cause fever and has been observed in five of the ten cases (50%) compared to infections caused by unknown etiology (16.81%) (*P* = 0.0197). No significant difference was observed in the incidence of vomit, diarrheal frequency, and stool routine examination (WBC count and OB text) between single HAstV infection and other virus infections or infections by unknown etiology. The median (IQR) of diarrheal frequency (times/day) in patients with single HAstV, single SaV, and other virus infections were 6.5 (4.75, 8.50), 3.50 (3.00, 7.00), and 5.00 (4.00, 8.00), although the difference was not statistically significant. On other hand, we did not find any difference in the clinical characteristics of patients with single HAstV/SaV infections and those showing coinfection with HAstV/SaV (Table 2).

### Nucleotide sequencing and phylogenetic analysis of HAstV and SaV

**Fig. 3:**
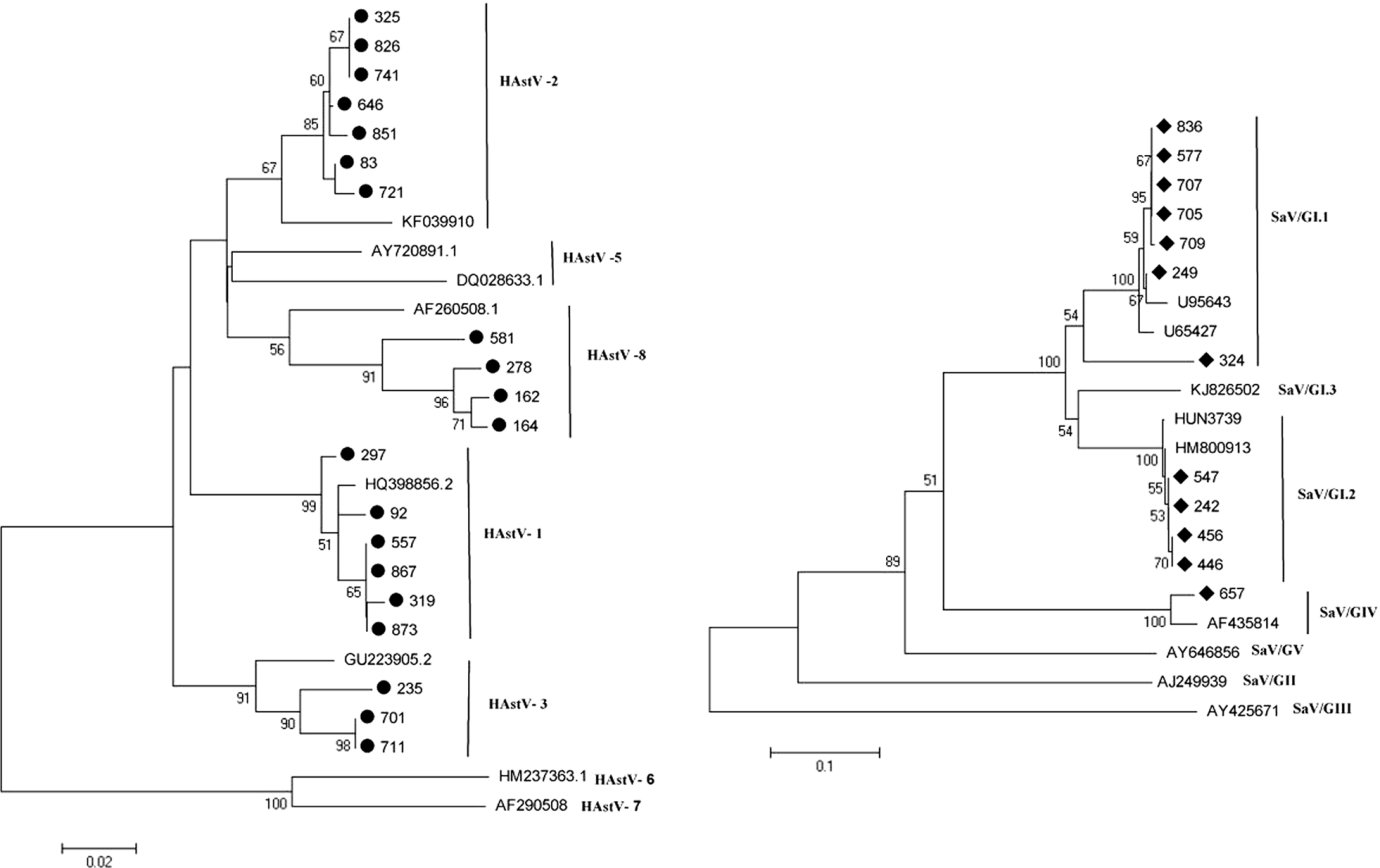
Phylogenetic trees of nucleotide sequences of 20 isolates of HAstV (●) and 12 isolates of SaV (◆). The trees were constructed from the partial nucleotide sequences of *ORF1a* of HAstV and the capsid region of SaV detected in Guangzhou city, China, from September 2013 to January 2016. The number on the branches indicate percentages and only those above 50% were shown. Reference strains of HAstV and SaV were selected from the GenBank under the accession number indicated in the text

A total of 20 HAstV and 12 SaV sequences were analyzed by phylogenetic analysis. Four genotypes (genotypes 1, 2, 3, and 8) of HAstV and three genotypes (GI.1, GI.2 and GIV) of SaV were identified (Fig. 3). HAstV-2 (35%, 7/20) was the dominant genotype, followed by HAstV-1 (30%, 6/20), HAstV-8 (20%, 4/20) and HAstV-3 (15%, 3/20). SaV GI.1 had the highest constituent ratio at 58.3% (7/12), followed by SaV GI.2 (33.3%, 4/12) and SaV GIV (8.3%, 1/12).

## Discussion

AGE is one of the most common disease around the world. However, other than rotavirus, norovirus and enteric adenovirus, knowledge about the impact of infections caused by HAstV and SaV is still much needed. In an effort to understand better the role of HAstV and SaV as a cause of AGE, a comprehensive survey on HAstV and SaV was carried out in Guangzhou, China. In-house multiplex RT-PCR and xTAG GPP, which had been validated for enteropathogens detection ability before, were used to detect HAstV, SaV and other enteropathogens associated with AGE [8, 14].

The detection rate of HAstV in people with AGE was 3.0% in the present study, which is similar to that previously reported in shanghai (5.22%), Thailand (2.6%), Asian Russia (2.8%) and Germany (5.0%), but was lower than the mean incidence worldwide of 11.0% [13, 15–18]. The SaV positive rate was 1.8% in Guangzhou, which is lower than that observed in US (7.0%) and Canada (9.5%) [19, 20]. Coinfection was found in 50% of the HAstV-positive samples and 16.7% of the SaV-positive samples in our study. Using real-time RT-PCR, other studies have reported a higher rate of coinfection with HAstV in viral gastroenteritis, ranging between 77 and 80% [21, 22]. Zhuo et al. reported a 35% prevalence of coinfection with SaV in Western Canada over a 4-year period [20]. Our slightly lower positivity can be explained by differences in the sample size, geographic location, and detection method. Norovirus and rotavirus were the most frequently detected enteropathogens responsible for coinfection in our study, which is in agreement with earlier reports [21–24]. It can be suggested that coinfection with HAstV or SaV is not rare. Our data also confirmed HAstV and SaV as the common enteropathogens responsible for AGE in Guangzhou. However, PCR can pick up free nucleic acid or long-term intermittent and asymptomatic shedding of HAstV, SaV or other enteropathogens in patients. A positive detection does not always correspond to active infection. Further investigation of the viral load and pathogenic mechanism is required.

SaV was detected most commonly in 1–5-year age group (5.97%), which is in line with those reported previously, ie most of the HAstV, SaV, rotavirus, norovirus, and enteric adenovirus infections occurred predominantly in infants and children < 5 years of age [21–24]. However, in contrast to the studies above, the prevalence of HAstV was the highest in 15–59-year age group (11.90%) in this study. Our data also showed that the median age of patients with the single HAstV infection was higher than that of the patients with other virus infections (*P* = 0.0476). 10 of total 20 cases with HAstV infection reported in 15–59-year age group, suggesting that adult may be susceptible to HAstV, not just the very young.

December to February the following year is the winter season in China. In our study, the highest incidence of HAstV occurs in winter (February) and that of SaV in early spring (March). Similar to several other studies of viral gastroenteritis conducted in India, Australia, Italy and Shanghai, HAstV infection was more frequent during the cold weather period in Guangzhou [18, 23, 25, 26]. SaV infection has been found mainly in the cold season [20, 27, 28]. However, the present study showed that SaV infection was more common during winter to early spring (February to March) in Guangzhou.

HAstV and SaV infections cause low-to-moderate degree of AGE with vomiting, fever, anorexia, abdominal pain, and dehydration [13, 29]. In our study, all HAstV- and SaV-positive cases were associated with acute watery gastroenteritis. Fever and vomiting were reported in 30% and 35% of HAstV infections and in 8.3% and 16.6% of SaV infections, respectively. In contrast to the study that reported greater diarrhea and fever prevalence, longer duration and greater intensity of diarrhea in HAstV infection compared to that in norovirus infections, our data did not show any significant difference in diarrhea frequency, fever prevalence between single HAstV infection and other virus infections [30]. Some reports suggest that coinfection may lead to more severe diarrhea [31, 32]. However, we could not find any significant difference in the specific clinical severity of AGE between single HAstV/SaV infections and mixed HAstV/SaV infections, which is in accordance with the reports from India and other countries [30, 33, 34]. In one case of *Campylobacter, Salmonella*, rotavirus, and SaV coinfection, no fever or vomiting was observed; in addition, the test for the presence of white/red blood cell test in the stool was negative, with low degree of gastroenteritis symptoms.

HAstV-1 is the most common HAstV genotype circulating worldwide [35–37]. However, predominant genotypes may change in different geographical locations, as has been reported in cases such as HAstV-2-associated childhood viral gastroenteritis outbreaks in Colombia [38]. In our study, six cases of HAstV-1, seven cases of HAstV-2, three cases of HAstV-3, and four cases of HAstV-8 were noted. HAstV-1 and HAstV-2 are predominant genotypes of HAstV in Guangzhou. SaV GI is the most common genogroup, while SaV GI.1 is the most common genotype globally [15, 24, 39]. In our study, 91.7% of the detected SaV cases belonged to GI, which is in accordance with literature. The most prevalent SaV genotype was GI.1 (58.4%), followed by GI.2 (33.3%) and GIV (8.3%).

## Conclusion

Although this study has established HAstV and SaV as the common causes of AGE in Guangzhou, the true etiologic agent could not be determined with certainty because of the high proportion of coinfection. Hence, further investigation of the viral load or unrecognized agents is required. The results of this study may provide further epidemiological and molecular information about HAstV and SaV strains causing AGE.

## Data Availability

The datasets analysed during the current study available from the corresponding author on reasonable request.
